# Moral foundations of pro-choice and pro-life women

**DOI:** 10.1007/s12144-023-04800-0

**Published:** 2023-06-01

**Authors:** Mariola Paruzel-Czachura, Artur Domurat, Marta Nowak

**Affiliations:** 1 Institute of Psychology, University of Silesia in Katowice, Grazynskiego 53, 40-126 Katowice, Poland; 2 Penn Center of Neuroaesthetics, Goddard Laboratories, University of Pennsylvania, 3710 Hamilton Walk, Philadelphia, PA 19104 USA; 3 Healio Institute of Psychotherapy in Katowice, Bazantow 35, 40-668 Katowice, Poland

**Keywords:** Abortion, Moral foundations, Moral judgment, Conservatism, Religious practice

## Abstract

Opinions on abortion are more polarized than opinions on most other moral issues. Why are some people pro-choice and some pro-life? Religious and political preferences play a role here, but pro-choice and pro-life people may also differ in other aspects. In the current preregistered study (*N* = 479), we investigated how pro-choice women differ in their moral foundations from pro-life women. When the Moral Foundations Questionnaire (MFQ) was applied (i.e., when declared moral principles were measured), pro-life women scored higher than pro-choice women in loyalty, authority, and purity. However, when women were asked about moral judgments indirectly via more real-life problems from the Moral Foundations Vignettes (MFV), pro-choice women scored higher than pro-life women in emotional and physical care and liberty but lower in loyalty. When we additionally controlled for religious practice and political views, we found no differences between groups in declaring moral foundations (MFQ). However, in the case of real-life moral judgments (MFV), we observed higher care, fairness, and liberty among pro-choice and higher authority and purity among pro-life. Our results show intriguing nuances between women pro-choice and pro-life as we found a different pattern of moral foundations in those groups depending on whether we measured their declared abstract moral principles or moral judgment about real-life situations. We also showed how religious practice and political views might play a role in such differences. We conclude that attitudes to abortion “go beyond” abstract moral principles, and the real-life context matters in moral judgments.

**Graphical abstract:**

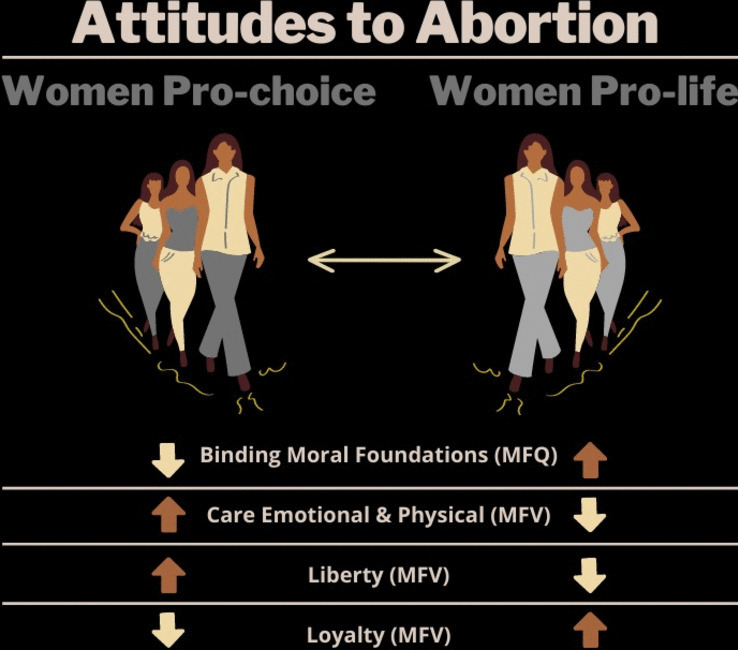

**Supplementary information:**

The online version contains supplementary material available at 10.1007/s12144-023-04800-0.

Banning the termination of pregnancy due to severe and irreversible damage to the fetus was approved in October 2020 in Polish legislation, which turned out to be one of the most restrictive abortion laws in Europe. Similarly, some American states have enacted new abortion restrictions in 2021 and 2022. Those changes provoked protests and showed how one moral issue, i.e., “the abortion problem”, may polarize societies. We already know that opinions on abortion were “always” polarized (Foot, 1967; Singer, 2011; Thomson, 1971; Watt, 2017), and they are also very stable (Kiley & Vaisey, 2020). Moreover, they are more polarized than opinions on most other moral issues (Baldassarri & Park, 2020; DiMaggio et al., 1996; Jones, 2018). Nevertheless, why are some individuals pro-life or pro-choice, and what characterizes those two groups?

Past research tried to answer these questions showing mainly how religiosity and political preferences shape the attitude to abortion. More religious and conservative people are usually more willing to declare pro-life (Barkan, 2014; Fiorina, 2017; Jędryczka et al., 2022). The abortion problem is indeed strongly related to religion, and religion is strongly related to politics (Jelen & Wilcox, 2003; Malka et al., 2012). When the religion is against abortion, for example, in the case of the Roman Catholic Church, the followers are usually pro-life (Jonason et al., 2022).

But moral judgments related to abortion are based mainly on the strength or salience of personal values (Rilling & Sanfey, 2011; Schwartz, 2007; Spicer, 1994), and religious or political preferences are just the indicators of those values (Koleva et al., 2012). That is probably why religious and political preferences were commonly studied as predictors of attitudes to abortion. However, one can approach the abortion problem from another perspective, i.e., look at it through the lens of moral foundations theory (Graham et al., 2018; Graham & Haidt, 2012). This theory, in its latest version, postulates six moral foundations, i.e., care, fairness, liberty (so-called three individualizing foundations), loyalty, authority, and purity (so-called three binding foundations) (Clifford et al., 2015).

## The moral foundations theory and the abortion problem

Moral foundations theory (Graham et al., 2009, 2013, 2018; Haidt, 2001) was proposed to explain why moral beliefs vary so widely across cultures yet still show many similarities and recurrent themes (Haidt & Graham, 2007). The first version of the theory posited that people differ in evaluating the importance of five moral foundations: care, fairness, loyalty, authority, and purity (Graham et al., 2018). The *care* foundation (the opposite of *harm*) relates to feeling empathy for the pain of others. *Fairness* (the opposite of *cheating*) concerns sensitivity to justice, rights, and equality. *Loyalty* (the opposite of *betrayal*) refers to the tendency to form coalitions and feel proud of being a group member. *Authority* (the opposite of *subversion*) relates to a preference for hierarchical social interactions and feeling respect for, or fear of, people in a higher social position. Finally, the *purity* (previously termed *sanctity*) foundation (the opposite of *degradation*) refers to a propensity to exhibit disgust in response to incorrect behavior and reflects individual differences in concerns for the sacredness of values (Koleva et al., 2012). Care and fairness are *individualizing* foundations. They are person-centered and focus on protecting individuals, whereas loyalty, authority, and purity are conceptualized as *binding* foundations because they focus on preserving one’s group as a whole (Graham et al., 2009, 2013, 2018). In the last modification of the theory, the sixth moral foundation of *liberty* was added (Graham et al., 2018). A higher level of liberty means a higher need to be free in our choices and behaviors. Liberty is also an individualizing moral foundation.

Only two studies tested how moral foundations might be related to attitudes to abortion. In the first study, Koleva and colleagues (Koleva et al., 2012) found that purity (measured by the Moral Foundations Questionnaire – MFQ of Graham and colleagues) predicted being pro-life. Specifically, they conducted two studies involving thousands of participants and a variety of moral issues (among them: the abortion problem), and they tested if the endorsement of five moral foundations may predict judgments about these issues, also testing the role of political ideology (measured by self-assessment on a scale from *very liberal* to *very conservative*), age, gender, religious attendance (i.e., frequent church attendance), and interest in politics. Regarding the abortion problem, only purity predicted attitude to abortion, next to conservative ideology and frequent church attendance. Despite the relevance of this result, this study focused only on declared preferences for moral foundations (i.e., used MFQ). We already know that those abstract preferences or principles do not always predict real-life decisions (Bostyn et al., 2018; Schein, 2020). For example, regarding the abortion problem, it was already found that some people, despite declaring they are against abortion, decided to help a close friend or family member seeking an abortion (Cowan et al., 2022). That is why we also need to study moral foundations more indirectly, for example, by asking about moral decisions close to real life. Additionally, Koleva and colleagues did not test the relevance of the liberty foundation, which was later added to the MFT (Clifford et al., 2015; Graham et al., 2018). Moreover, they tested only general attitudes to abortion (for example, not measuring the possible impact of the abortion law on the participants or their close others). Lastly, they conducted the study before the latest law changes in 2020–2022, which could also impact attitudes toward such an important social issue.

In the second study, Jonason and colleagues (2022) asked 255 women and men from Poland about their attitudes toward Poland’s ban on abortion. They showed that Catholics were higher on binding moral foundations (measured via MFQ) than non-Catholics and that Catholics perceived the new situation in Poland with less negativity, which led them to support the ban more than non-Catholics. These results are consistent with past findings, as generally, being religious and conservative is related to being pro-life, and religiosity and conservatism turn out to be linked to binding moral foundations (Kivikangas et al., 2021; Saroglou & Craninx, 2020). Despite the relevance of this study, it also focused only on declared moral foundations (i.e., MFQ) and did not measure liberty as a new moral foundation (Clifford et al., 2015; Graham et al., 2018). Moreover, it focused mainly on attitudes toward Poland’s recent ban on abortion. Finally, the two studies mentioned above analyzed the general population, so it is hard to make general conclusions about the *differences* between pro-choice and pro-life. One possible way to study this issue deeply could be by studying two samples of individuals who clearly define themselves as pro-choice or pro-life. We aimed to do this in the current research.

## The current research

We aimed to provide deeper insights into the moral foundations among pro-choice and pro-life individuals. We wished to build on past work (Jonason et al., 2022; Koleva et al., 2012) in six ways:

1. we used two measures of moral foundations that could allow more general conclusions about the differences between being pro-life and pro-choice as they measure moral foundations directly (MFQ) and indirectly (MFV). Specifically, we measured moral foundations not only by asking about the declaration of moral preferences (declared the importance of and attitude to abstract moral principles) using MFQ (Graham et al., 2009) but also by measuring participants’ assessment of immoral actors in concrete, real-life scenarios using MFV (Clifford et al., 2015). Measuring declarative abstract moral principles with MFQ makes sense; nevertheless, abortion is a common real-life problem involving concrete actions and choices to be made (Cowan et al., 2022; Maddow-Zimet et al., 2021). Because MFQ relies on respondents’ rating of abstract principles, it is tough to say anything about respondents’ moral judgment of concrete scenarios (Clifford et al., 2015). Moreover, those abstract principles do not always predict real-life decisions (Bostyn et al., 2018; Schein, 2020), e.g., some people, despite being against abortion (declaration of abstract principle), decide to help a close friend or family member who is seeking an abortion (Cowan et al., 2022). That is why we used MFV, an indirect measure of moral foundations based on real-life situations;
2. by using MFV, we measured the new moral foundations of liberty, and to our best knowledge, we are the first to test the role of this foundation in the abortion problem;
3. by using MFV, we were able to measure two types of care foundation, i.e., emotional and physical care, so this way, we could test the sensitivity to emotional or physical harm in our sample;
4. we narrowed the sample to women. We did it for obvious biological reasons, i.e., women are more directly affected by the abortion rule than men. Past studies also show that our attitudes may be stronger if an object or issue may impact our lives more directly (Albarracín, 2021);
5. we decided to test two groups of women (i.e., pro-life and pro-choice). Past research (Jonason et al., 2022; Koleva et al., 2012) did not study such opposite groups; by this design, we could look for the clear differences between them;
6. we measured attitudes to abortion in more detail than in past studies (Jonason et al., 2022; Koleva et al., 2012). Specifically, we asked women about their attitude to abortion in three ways: by direct question whether they are pro-choice or pro-life, by asking about their views on four detailed issues concerning the new abortion law in Poland, and by using a scale that helped us to measure Full and Conditional Abortion Support (see Measures section).

### Hypothesis

Following past research (Jonason et al., 2022), we hypothesized that pro-life women would have higher levels of binding moral foundations than pro-choice women. Because moral foundations measured by MFQ and MFV correlated positively in past research (Clifford et al., 2015), we expected to observe the same pattern of results for both of them.

## Study

The Research Ethics Committee of the University of Silesia in Katowice accepted the current study. The materials, data, and code are available at https://osf.io/793cr/?view_only=None. The study was preregistered at https://aspredicted.org/i9fa8.pdf. We report all measured variables in this study.

### Method

#### Participants and procedure

We preregistered a survey with a sample of at least *N* = 300 respondents, *n* = 150 women pro-choice, and *n* = 150 women pro-life. Using G*Power 3.1.9.7 software suggested that we need to recruit two independent groups of ca. 150 participants, assuming alpha error probability of 0.05, power of 0.8, and low-to-medium effect size of 0.33 (of differences between groups on a dependent variable in two independent group comparisons). Because participants’ membership to one of two groups would be defined post hoc – based on the dichotomous question about support for abortion – and the allocation ratio to the groups was hard to predict a priori, we preregistered that if we collect more data in any of the two expected subsamples, we will include them in the analyses. We stopped the data collection when the smaller group had *n* = 150.

Our online study was conducted during a specific time in Poland in 2021, just after the Polish government introduced the new abortion law. We want to highlight that it was a stormy time when many people went on the streets to express their support for women’s rights, despite the COVID-19 pandemic, so despite that, their lives were directly in danger. Like the study by Jonason and colleagues (2022), contrary to Koleva and colleagues’ (2012) study, we asked about a real-life problem, as abortion was the main topic in media, hospitals, homes, etc.

Women were invited to an anonymous online survey in Qualtrics using the snowball method via the University of Silesia’s website and social media platforms. Five hundred sixteen participants took part in the study. All participants had Polish nationality and spoke the Polish language. We excluded participants who did not agree to participate in the study after reading the instruction (*n* = 6), did not answer attention check questions (*n* = 3), and one man from the sample. We also excluded participants (*n* = 27) with too short (less than 3:30 min.) or too long (more than 28 min.) survey completion times, defined by logarithms outside the interquartile range of [*Q*1–1.5 *IQR*, *Q*3 + 1.5 *IQR*]^1^.

The analyzed sample consisted of 479 women, split into two groups: pro-choice women (*n* = 332, *M*_age_ 26.34, *SD* = 7.53) and pro-life women (*n* = 147, *M*_age_ 27.84, *SD* = 7.20). Among pro-life women, *n* = 123 (83.7%) declared being Catholics, *n* = 11 (7.5%) reported being atheists, and *n* = 13 (8.8%) declared being other than Catholics (i.e., Buddhists, Protestants, other and not specified). Among pro-choice women, *n* = 158 (47.6%) reported being Catholics, *n* = 155 (46.7%) declared being atheists, and *n* = 19 (8.8%) declared being other than Catholics (Buddhists, Judaists, Orthodox Catholics, Protestants, other and not specified). However, it is worth noting that 177 (53.3%) pro-choice women practiced religion, and 11 (7.5%) pro-life women were not religious.

##### Group check

Our two groups were distinguished by asking women if they were pro-choice or pro-life. However, to ensure that women correctly divided themselves as pro-choice or pro-life, we asked them about more detailed attitudes to abortion (see section Measures).

#### Measures

##### Attitude to abortion

Women were asked about their attitudes to abortion in three ways. First, respondents answered a single question about whether they were pro-choice or pro-life (“If you had to define your own attitude towards abortion clearly, you are: pro-choice/pro-life”). This question was used to identify the two subsamples. Second, the participants expressed their views on four detailed issues concerning the new abortion law in Poland. The first question, “What is your attitude to the verdict issued by the Constitutional Court?” was answered on a scale from 1 (*I definitely do not support*) to 7 (*I definitely do support*) (variable: Attitude to New Rule in Table 1). The other three questions were about the potential impact of a new law on them personally (variable: Personal Influence in Table 1), on their close others (variable: Influence on Close Others in Table 1), and generally on other women (variable: General Influence in Table 1) and they were answered on a scale from 1 (*definitely negative*) to 7 (*definitely positive*). Third, participants read six statements about attitudes to abortion and evaluated to what extent they agreed with the statements using a scale from 1 (*I disagree*) to 5 (*I agree*). The first three statements were: “I support the full right to abortion, which is the inalienable right of every woman”, and “Abortion is a woman’s personal matter, and no one else can decide for her whether she should have an abortion or not”, “Abortion should be allowed regardless of the reason”. Averaged answers for these three statements created the index of Full Abortion Support (Cronbach *α* = 0.92). Similarly, the following three statements: “Abortion should be allowed only if the pregnancy threatens the life or health of the mother ”, “I support the introduction of the full right to abortion, but only up to the 12th week of pregnancy”, and “Abortion is allowed only when we are sure that the child will be born with a genetic defect” were to create the Conditional Abortion Support index, however, due to its low consistency (*α* = 0.11), we decided to analyze them separately.

##### Moral Foundations Questionnaire

We used a Polish adaptation (Jarmakowski-Kostrzanowski & Jarmakowska-Kostrzanowska, 2016) of the Moral Foundations Questionnaire (MFQ; Graham et al., 2009) to measure the degree to which the participants endorsed five sets of moral intuitions (i.e., care, fairness, loyalty, authority, and purity) in moral decision-making. The scale consists of 30 items that measure the moral foundations in two ways: a relevance subscale (15 items) showing how important each one of the moral foundations is for a person, and a judgments subscale (15 items), which measures the extent to which people agree with various moral opinions connected with the different moral foundations. An example item for care is “It can never be right to kill a human being”; for fairness: “When the government makes laws, the number one principle should be ensuring that everyone is treated fairly”; for loyalty: “People should be loyal to their family members, even when they have done something wrong”; for authority: “Men and women each have different roles to play in society”; and for purity: “People should not do things that are disgusting, even if no one is harmed”. A 1 to 6 response scale was used for all items, where 1 was *not at all relevant* or *strongly disagree*, and 6 was *extremely relevant* or *strongly agree*. Responses were averaged to give an overall score for each foundation. Cronbach alphas were found to be moderate for care (*α* = 0.61) and fairness (*α* = 0.56) and high for loyalty (*α* = 0.77), authority (*α* = 0.76), and purity (*α* = 0.82).

##### Moral Foundations Vignettes

It measures moral foundations based on evaluating other people’s behavior violating them (MFV; Clifford et al., 2015). The randomized set of 21 vignettes was used in our study, three vignettes per moral foundation. Apart from using five classic moral foundations, it includes a liberty foundation and two types of care, i.e., sensitivity to emotional harm to humans or non-human animals (care emotional) and sensitivity to physical harm to humans or non-human animals (care physical). An example item for care emotional is “You see a woman commenting out loud about how fat another woman looks in her jeans”; for care physical: “You see a zoo trainer jabbing a dolphin to get it to entertain his customers”; for fairness: “You see a boy skipping to the front of the line because his friend is an employee”, for liberty: “You see a man forbidding his wife to wear clothing that he has not first approved”; for loyalty: “You see the US Ambassador joking in Great Britain about the stupidity of Americans” [changed into Polish Ambassador in Germany]; for authority: “You see an employee trying to undermine all of her boss’ ideas in front of others”; for purity: “You see an employee at a morgue eating his pepperoni pizza off of a dead body”. The 5-point scale was used from 1 (*not at all wrong*) to 5 (*extremely wrong*). We did translation-back-translation of MFV (see Materials at OSF). Cronbach alphas were satisfactorily high for care emotional (*α* = 0.88), fairness (*α* = 0.71), liberty (*α* = 0.72), authority (*α* = 0.71), and loyalty (*α* = 0.76), and moderate for care physical (*α* = 0.68) and purity (*α* = 0.56).

##### Religious practice

Participants were asked to evaluate their level of practicing religion on a scale from 1 (*I don’t practice at all*) to 8 (*I am a very practicing person*). Additionally, we asked about which type of religion they practiced (if they practiced any).

##### Political views

We asked participants two questions about their political views, one related to economic issues (“Please indicate on the following scale your political views relating to economic issues”) on a scale from 0 (*State participation should be very small*) to 7 (*State participation should be very high*), and the other one related to social issues (“Please indicate on the following scale your political views relating to social, cultural issues”) on a scale from 0 (*very conservative*) to 7 (*very liberal*).

## Results

Descriptive statistics and differences between pro-choice and pro-life women in religious practice, political views, and attitudes to abortion are shown in Table 1. The two groups differed (Welch t-tests) significantly in practicing religion (lower among pro-choice) and political views on social issues (higher liberal views among pro-choice), but there was no difference between the groups in views on economic issues. Pro-choice and pro-life women differed in full support for abortion, meaning the two groups differed in their extreme views on abortion. Moreover, pro-life women had stronger beliefs that the new abortion rule in Poland would positively impact themselves personally, their close others, and women in general. In contrast, pro-choice women believed more that the new law would harm all women, themselves, and their close others.

Regarding conditional support, women pro-life agreed more with two statements allowing abortion conditionally when the pregnancy threatens the mother’s life or health and when one is sure that the child will be born with a genetic defect. Women pro-choice agreed more with the third statement allowing the right to abortion until the 12th week of pregnancy (Table 1).

Summing up, the observed differences, especially in full support of abortion, show that women accurately classified themselves into one of the two groups, and we can be sure that the groups indeed evaluate abortion from different standpoints (however, see the limitation section for elaboration on improving such classification).

**Table 1:** Descriptive statistics and differences between pro-choice and pro-life women in religious practice, political views, and attitudes to abortion

Measures	Pro-Choice *N* = 332		Pro-Life *N* = 147					
	*M*	*SD*	*M*	*SD*	*t*	*df*	*p*	*Cohen’s d*
Religious Practice [1–8]	2.48	1.84	5.67	2.16	– 15.59	244.4	< 0.001	– 1.64
Economic Issues [0–7]	3.89	1.59	3.97	1.78	– 0.46	254.1	0.647	– 0.05
Social Issues [0–7]	5.64	1.37	3.73	1.80	11.45	224.1	< 0.001	1.26
Full Abortion Support [1–5]	4.42	0.86	1.83	1.09	25.39	230.1	< 0.001	2.75
Conditional Support, item1 [1–5]	1.74	1.19	3.20	1.53	-10.33	228.6	< 0.001	-1.12
Conditional Support, item2 [1–5]	3.33	1.42	1.55	1.07	15.10	363.0	< 0.001	1.35
Conditional Support, item3 [1–5]	1.72	1.22	2.01	1.23	-2.39	277.5	0.018	-0.24
Attitude to New Rule [1–7]	1.13	0.50	3.71	2.25	– 13.75	152.5	< 0.001	– 1.96
Personal Influence [1–7]	1.60	0.90	3.89	1.57	– 16.60	189.6	< 0.001	– 2.00
Influence on Close Others [1–7]	1.48	0.81	3.53	1.63	– 14.46	179.1	< 0.001	– 1.82
General Influence [1–7]	1.21	0.49	3.06	1.91	– 11.60	154.7	< 0.001	– 1.63

The numbers in brackets are the variable’s scales

Next, we run analyses to see if moral foundations measured in two ways (i.e., MFQ and MFV) correlated. As shown in Table 2, we received positive correlations among analogous dimensions of moral foundations, replicating past results (Clifford et al., 2015).

**Table 2:** Pearson correlations between moral foundations measured by MFQ and MFV

	MFQ: Care	MFQ: Fairness	MFQ: Loyalty	MFQ: Authority	MFQ: Purity
MFV: Care Emotional	0.245^***^	0.306^***^	0.096^*^	0.024	0.075
MFV: Care Physical	0.257^***^	0.226^***^	0.032	− 0.037	0.004
MFV: Fairness	0.118^**^	0.313^***^	0.112^*^	0.090^*^	0.116^*^
MFV: Liberty	0.160^***^	0.306^***^	0.069	− 0.074	− 0.005
MFV: Authority	0.110^*^	0.236^***^	0.403^***^	0.395^***^	0.411^***^
MFV: Loyalty	0.112^*^	0.177^***^	0.506^***^	0.471^***^	0.432^***^
MFV: Purity	0.210^***^	0.190^***^	0.301^***^	0.269^***^	0.418^***^

^*^p < .05, ^**^p < .01, ^***^p < .001 two-sided.

Finally, we run analyses to see if the groups differ in moral foundations (ANOVA) and when controlling for political views and religious practice simultaneously (ANCOVA).

### Preregistered analyses

#### Do pro-choice and pro-life women differ in moral foundations?

Yes. As shown in Table 3, when we analyzed differences between groups (ANOVA) using the classical measure of moral foundations (i.e., MFQ), we found that pro-life women had significantly higher binding foundations than pro-choice women, i.e., loyalty (medium effect size), authority (medium effect size), and purity (large effect size). We observed a different pattern of results when using the MFV (with small effect sizes for all results), a more indirect measure of moral foundations. For binding moral foundations, only loyalty seemed to play a role here, i.e., pro-life women had a higher level of loyalty than pro-choice women. However, pro-choice women had higher levels of both types of care (i.e., emotional and physical) and liberty than pro-life women. Fairness, authority, and purity did not differentiate those groups using MFV.

**Table 3:** Tests of effects in ANOVA and ANCOVA

	Descriptive Statistics		ANOVA		ANCOVA							
	Pro-Choice	Pro-Life	Attitude Toward Abortion		Attitude Toward Abortion		Political Views on Economic Issues		Political Views on Social Issues		Religious Practice	
	*M* (*SD*)	*M* (*SD*)	*F*(1,477)	*η*^2^_p_	*F*(1,474)	*η*^2^_p_	*F*(1,474)	*η*^2^_p_	*F*(1,474)	*η*^2^_p_	*F*(1,474)	*η*^2^_p_
MFV Care(emotional)	4.46 (0.73)	4.20 (0.88)	11.56^***^	0.024	8.36^**^	0.017	7.38^**^	0.015	0.16		0.44	
Care(physical)	4.60 (0.58)	4.36 (0.72)	14.04^***^	0.029	5.54^*^	0.012	6.23^*^	0.013	0.91		2.74	
Fairness	4.43 (0.62)	4.31 (0.64)	3.81		6.95^**^	0.014	1.41		2.94		0.24	
Liberty	4.34 (0.69)	4.00 (0.84)	21.84^***^	0.044	14.28^***^	0.029	1.69		2.65		2.25	
Authority	3.08 (0.89)	3.23 (1.04)	2.57		10.71^**^	0.022	3.97^*^	0.008	3.06		35.61^***^	0.070
Loyalty	3.24 (1.01)	3.52 (1.01)	7.86^**^	0.016	0.85		6.36^*^	0.013	11.50^***^	0.024	5.76^*^	0.012
Purity	3.90 (0.78)	3.96 (0.96)	0.51		4.68^*^	0.010	12.25^***^	0.025	4.41^*^	0.009	7.24^**^	0.015
MFQ Care	5.24 (0.56)	5.30 (0.55)	1.12		3.09		4.72^*^	0.010	2.71		0.1	
Fairness	4.95 (0.57)	4.87 (0.58)	2.00		0.45		8.47^**^	0.018	0.64		0.02	
Loyalty	3.15 (0.88)	3.57 (0.81)	24.29^***^	0.048	1.03		9.38^**^	0.019	28.75^***^	0.057	16.04^***^	0.033
Authority	2.84 (0.91)	3.42 (0.93)	39.95^***^	0.077	1.39		11.14^***^	0.023	64.68^***^	0.120	20.2^***^	0.041
Purity	3.12 (0.99)	4.17 (1.13)	106.48^***^	0.182	0.06		7.13^**^	0.015	49.48^***^	0.095	91.42^***^	0.162

^*^p < .05; ^**^p < .01; ^***^p < .001. The rows contain tests of one ANOVA with moral foundation as a dependent variable and attitude toward abortion as a factor, and one ANCOVA, extending the ANOVA with the set of covariates: religious practice, political views on economic issues, and political views on social issues

### Exploratory analyses

#### Do pro-choice and pro-life women differ in moral foundations when we control religious practice and political views?

Yes. When we controlled for political views and religious practice simultaneously (ANCOVA), we found no differences between groups regarding declared moral foundations (MFQ). However, in the case of real-life assessments (MFV), we observed the same pattern of results for care and liberty as when using ANOVA, but now loyalty did not differentiate these two groups. Additionally, we observed differences in fairness, authority, and purity in such a way that women pro-life had higher levels of those foundations than women pro-choice. All found effects were small.

## Discussion

Past research tried to explain attitudes to abortion mainly by looking into religious and political differences between pro-choice and pro-life people. However, attitudes to abortion may also be related to an individual’s moral views (Jędryczka et al., 2022; Jonason et al., 2022), and sometimes moral foundations may even be an as good predictor of attitudes to abortion as a religious practice or political conservatism (Koleva et al., 2012). In the current research, we looked into the problem of attitudes to abortion more deeply by studying, directly and indirectly, moral foundations among pro-choice and women pro-life women.

When we asked about moral foundations directly (using MFQ of Graham and colleagues, 2009), we confirmed our preregistered hypothesis that pro-life women have higher binding foundations than pro-choice women. This result is consistent with past findings (Jonason et al., 2022). However, we found a different pattern of results when measuring moral foundations indirectly, i.e., by MFV (Clifford et al., 2015). For binding foundations, only loyalty seemed to play a role here, i.e., pro-life women had a higher level of loyalty than pro-choice women. Regarding individualizing foundations, pro-choice women had higher care (physical and emotional) and liberty levels than pro-life women. Fairness, authority, and purity did not differentiate those groups when applying MFV.

Moreover, when we additionally controlled for religious practice and political views (ANCOVA), we found no differences in moral foundations between groups regarding declared moral foundations (MFQ). However, in the case of real-life assessments (MFV), we observed higher care and liberty among pro-choice (just like in ANOVA) and higher fairness, authority, and purity among pro-life. We conclude that religious practice and political views may explain differences between pro-choice and pro-life, but only in the case of declared moral foundations (MFQ) and not in MFV (when individuals make moral judgments about real-life behaviors). Because we found differences between pro-choice and pro-life women (whether we controlled religious practice or political views or not), we conclude that studying indirect moral judgments (i.e., using MFV) may reveal hitherto unknown “hidden” differences between pro-choice and pro-life women.

Specifically, our results show intriguing nuances in the problem of abortion as we found that pro-choice and pro-life women differ in declared abstract moral principles (MFQ) and sensitivity to violating those principles in real-life situations (MFV). On the one hand (i.e., when using the MFQ), women who were pro-life were the women who intensely cared about binding foundations, which was also related to their more vital religious practices and higher conservatism on social issues. It simply means that women who were pro-life cared more about binding foundations than pro-choice women, so they declared that they cared about being loyal, listening to authorities, and not violating the purity foundation, which is strictly related to religious sanctity (and indeed, this foundation’s one of the first names was even *sanctity*) (Graham et al., 2018). Indeed, past studies showed strong correlations between religion and binding moral foundations worldwide (Saroglou & Craninx, 2020) and conservative political preferences and binding foundations (Kivikangas et al., 2021). Similar associations were found between five moral foundations, religiosity, political preferences, and acceptance of the new abortion rule in Poland (Jonason et al., 2022) or between preference for group-based hierarchy and pro-life (Osborne & Davies, 2009). When we controlled for religious practice and political views, the differences between pro-choice and pro-life women disappeared, so we can conclude that – at least for declared abstract moral foundations – being religious and conservative plays a central role in the abortion problem.

On the other hand (i.e., when using the MFV), we showed that this is only one part of the story. We know it because when indirectly measuring preferences for moral foundations, the same women (i.e., pro-life) had higher levels of only loyalty foundation when compared to pro-choice women. The importance of loyalty to the abortion problem is consistent with theory and past findings (Jonason et al., 2022). Higher levels of loyalty are related to being more religious and conservative (Saroglou & Craninx, 2020). The more surprising result is that authority and purity foundations did not play an essential role in the abortion problem when measured indirectly. This result contradicted past findings when moral foundations were measured directly (Jonason et al., 2022). It may be related to a different approach to measuring moral foundations by MFQ and MFV. For example, purity is more directly connected to religiosity in MFQ than in MFV, and their operationalization is slightly different (Crone, 2022). We suspect it is the most reasonable explanation for finding no differences here. However, when we additionally controlled for religious practice and political views, we replicated the higher level of care and liberty among pro-choice, but we also found a higher level of fairness, authority, and purity among pro-life. Future researchers could try to explain those nuances more deeply, e.g., by conducting longitudinal studies or using more complex measurements of religiosity and political preferences. We observe inconsistent patterns of results for binding moral foundations measured via MFV, so we should be more tentative about the interpretation and conclusions from our study. We need more studies on this issue to understand why we observed such inconsistency.

Regarding the individualizing moral foundations (MFV), pro-life women scored lower in physical and emotional care and liberty foundations than pro-choice women (also when controlling for religious practice and political views). Regarding care, it simply means that pro-choice and pro-life women gave similar declarations about how important it is for them to care about others (MFQ). However, they differed in indirect measures of care in such a way that pro-choice women had higher levels of care than pro-life women (MFV). These results are the most intriguing for us. Women being pro-life sometimes argue that they care about all life, so abortion should be banned. Nevertheless, we did not find confirmation of this in empirical results. Surprisingly, those women who were pro-choice had higher levels of emotional and physical care than pro-life women. It means that when making moral decisions about other people, pro-choice women were more sensitive to violations of care foundation or, in other words: they disliked the suffering of others more than pro-life women. According to some approaches in moral psychology, the foundation of care is the most critical, and people make their moral judgments mainly based on a simple question: Is anyone hurt? (Gray et al., 2012; Schein & Gray, 2018). Future studies are needed to explain those differences in care, looking for possible sources of them, maybe in the levels of empathy (Zaki, 2018), moral identity (Aquino & Reed, 2002; Paruzel-Czachura & Blukacz 2021), moral absolutism (Vecina et al., 2016), or more general attitudes to violence (Vecina et al., 2015).

As MFQ does not allow measuring the liberty foundation, we only studied its level using the MFV, and we found that pro-choice women had a higher level of liberty than pro-life women. The importance of liberty is consistent with theoretical assumptions of being pro-choice (Foot, 1967; Singer, 2011; Thomson, 1971; Watt, 2017), and it is the first result confirming empirically that, indeed, being pro-choice is related to highlighting liberty when making moral decisions about what behavior is right or wrong.

Some individuals may say they are pro-life or pro-choice because of their religious or political preferences. Indeed, we found significant relations between stronger practicing of religion, conservative views on social issues, and being against abortion. However, we also found this may be too straightforward to describe this problem because there are atheists and believers in both groups of women, i.e., pro-choice and pro-life. We need more studies to understand the complex attitudes to abortion, for example, by studying only a sample of atheists. It is also worth highlighting again that past studies showed that moral foundations might be as good a predictor of attitudes to abortion as religious or political views (Koleva et al., 2012). Because of the importance of the abortion problem in our everyday lives, we need more studies to understand possible differences between pro-choice and pro-life people beyond simple explanations that abortion is just a matter of religion or politics.

Our study is not free from limitations. First, we tested only one sample. There is a possibility that different samples (e.g., from other cultural or religious backgrounds) would bring different results. We cannot know to what extent the results are dependent on the Polish context and the abortion protests, and this is a limitation that needs to be addressed in future research. We need replications of our study, especially in diverse samples, including countries where the abortion law changed, similar to Poland. Attitudes to abortion may be sensitive to changes in law, which made thousands of women protest for their rights on the streets in the case of Poland. Second, we did not study whether being pro-choice or pro-life is moderated by individual differences. For instance, attitudes or moral judgments may depend on personality (Pratto et al., 1994). Does personality matter for the abortion problem, and if yes, how? (Jonason et al., 2022). Third, we also did not study how situational factors may impact attitudes toward abortion, and some research shows that this issue is worth future investigations (Bago et al., 2022; Bilewicz et al., 2017). Fourth, two compared groups were identified based on a direct question about their position on pro-life or pro-choice. To cope with false self-identification, we asked additional questions about attitudes toward the abortion problem and the new law in Poland. Admittedly, we confirmed that women correctly assigned themselves to the group for or against abortion (see results: group check). However, we did not avoid the problem related to the situation that some participants who claimed to be pro-life or pro-choice had more mixed feelings about the rest of the questions. We conducted additional analyses to understand this issue more deeply (Supplementary Materials). Specifically, we presented the percentages of participants’ answers within the two groups on the six statements expressing full or conditional support for abortion (Table S1). This table shows that most participants correctly assigned themselves to the group. However, there were participants whose feelings were mixed. Moreover, we conducted the hierarchical cluster analysis on the three statements expressing full support for abortion and observed that some participants do not belong to the two obtained clusters (Table S2). Because we did not preregister to drop such participants out, we did not do it. However, we recommend implementing better control of this issue in future studies to ensure that such groups are created properly. Fifth, we measured religious practice and political views by only single items. In future studies, researchers could use more complex measures of those variables, e.g., the Centrality of Religiosity Scale (Huber & Huber, 2012) or the Resistance to Change-Beliefs Scale (White et al., 2020). Sixth, it is worth noticing that the correlations between the factors estimated through the MFQ and the MFV are mediocre, or some correlate not exactly as the theory would expect. For instance, MFV authority correlates with MFQ fairness. Perhaps different results with MFQ and MFV might be caused by the imprecision of the instruments in measuring moral foundations. Lastly, there is also a possibility that different results would be obtained in non-WEIRD samples (that are White, Educated, Industrialized, Rich, and Democratic) (Henrich et al., 2010), as some research has suggested different patterns of moral judgments in non-WEIRD samples (e.g., Smith & Apicella 2022; Sorokowski et al., 2020; Turpin et al., 2021; Workman et al., 2022). Despite all the above limitations, we believe that because of our topic’s theoretical and practical relevance, our study brings an important puzzle to understanding polarization regarding the abortion problem.

## Conclusions

We conclude that to understand the attitudes to abortion more fully, we must go beyond abstract moral declarations. Our research demonstrates that pro-choice and pro-life women differed in moral foundations when (a) they revealed abstract moral foundations (pro-life women cared more about loyalty, authority, and purity than pro-choice women) and (b) when they made moral judgments closed to real-life problems (e.g., pro-choice women were more concerned than pro-life women when the foundations of emotional and physical care and liberty were violated). Concerning the latest restrictions on abortion in many places worldwide, discussions about the abortion problem have become more common in our everyday lives. This issue touched many people so much that it sparked massive protests. Hence, it is essential that people are aware of these differences between pro-choice and pro-life women, and we definitely need more studies on this topic.

## Supplementary information

Below is the link to the electronic supplementary material.

ESM 1 (DOCX 24.2 KB)

## Data Availability

The materials, data, and code are available at https://osf.io/793cr/?view_only=None. The study was preregistered at https://aspredicted.org/i9fa8.pdf.

## Footnotes

1 We did not pre-register dropping those participants out. However, when we repeated the analyses for the full sample, we observed the very similar values of Cronbach alphas, the same pattern of correlations and differences between groups, and similar p-values in the performed statistical tests.
