# Supplementary material for: Moral foundations of pro-choice and pro-life women
Source: Curr Psychol. 2023 Jun 1:1–11. Online ahead of print. doi: 10.1007/s12144-023-04800-0 (PMC10233192; doi:10.1007/s12144-023-04800-0)
Supplement: Supplementary file 1 — (DOCX 24.2 KB) [file 12144_2023_4800_MOESM1_ESM.docx]

**Supplementary Materials**

**Table S1**

*The Percentages of Participants’ Answers Within the two Groups on the Three Statements Expressing Full or Conditional Support for Abortion*

| Full (F) and Conditional (C) Support Statements | Attitude Toward Abortion | 1  (*I disagree*) | 2 | 3 | 4 | 5  (*I agree*) | 1 & 2 | 4 & 5 |
| --- | --- | --- | --- | --- | --- | --- | --- | --- |
| 1. I support the full right to abortion, which is the inalienable right of every woman. F | pro-choice | 2.1 | 5.1 | 2.4 | 19.9 | 70.5 | 7.2 | **90.4** |
|  | pro-life | 64.6 | 18.4 | 4.1 | 7.5 | 5.4 | **83.0** | 12.9 |
| 2. Abortion is a woman’s personal matter, and no one else can decide for her whether she should have an abortion or not. F | pro-choice | 0.6 | 4.5 | 0.3 | 11.7 | 82.8 | 5.1 | **94.5** |
|  | pro-life | 44.9 | 20.4 | 3.4 | 13.6 | 17.7 | **65.3** | 31.3 |
| 3. Abortion should be allowed regardless of the reason. F | pro-choice | 8.1 | 10.5 | 6.3 | 19.0 | 56.0 | 18.6 | **75.0** |
|  | pro-life | 80.3 | 8.8 | 4.1 | 3.4 | 3.4 | **89.1** | 6.8 |
| 4. Abortion should be allowed only if the pregnancy threatens the life or health of the mother. C | pro-choice | 64.5 | 15.7 | 6.3 | 8.7 | 4.8 | **80.2** | 13.5 |
|  | pro-life | 21.1 | 17.0 | 9.5 | 25.2 | 27.2 | 38.1 | **52.4** |
| 5. I support the introduction of the full right to abortion, but only up to the 12th week of pregnancy. C | pro-choice | 14.8 | 16.6 | 17.8 | 22.6 | 28.3 | 31.4 | **50.9** |
|  | pro-life | 73.5 | 10.9 | 6.1 | 6.1 | 3.4 | **84.4** | 9.5 |
| 6. Abortion is allowed only when we are sure that the child will be born with a genetic defect. C | pro-choice | 66.3 | 15.1 | 4.8 | 7.8 | 6.0 | **81.4** | 13.8 |
|  | pro-life | 51.0 | 17.0 | 15.0 | 13.6 | 3.4 | 68.0 | 17.0 |

**Table S2**

*The Hierarchical Cluster Analysis on the Three Statements Expressing Full Support to Abortion: Frequencies and Percentages of the Assignment to the two Analyzed Groups (Women Pro-Choice and Women Pro-Life) Within two Clusters*

| Cluster | Attitude Toward Abortion | | | | Total | |
| --- | --- | --- | --- | --- | --- | --- |
|  | Pro-Choice | | Pro-Life | |  |  |
|  | *N* | % | *N* | % | *N* | % |
| 1 | 323 | 86,1 | 52 | 13,9 | 375 | 100 |
| 2 | 9 | 8,7 | 95 | 91,3 | 104 | 100 |
| Total | 332 | 69,3 | 147 | 30,7 | 479 | 100 |
